# Impact of biodiversity and seasonality on Lyme-pathogen transmission

**DOI:** 10.1186/1742-4682-11-50

**Published:** 2014-11-28

**Authors:** Yijun Lou, Jianhong Wu, Xiaotian Wu

**Affiliations:** Department of Applied Mathematics, The Hong Kong Polytechnic University, Hung Hom, Kowloon, Hong Kong; Department of Mathematics and Statistics, York University, Toronto, Ontario M3J1P3 Canada; Centre for Disease Modelling, York University, Toronto, Ontario M3J1P3 Canada; Department of Applied Mathematics, Western University, London, Ontario N6A5B7 Canada

**Keywords:** Seasonal tick population, Lyme disease, Host diversity, Climate, Threshold, Dilution effect, Amplification effect

## Abstract

Lyme disease imposes increasing global public health challenges. To better understand the joint effects of seasonal temperature variation and host community composition on the pathogen transmission, a stage-structured periodic model is proposed by integrating seasonal tick development and activity, multiple host species and complex pathogen transmission routes between ticks and reservoirs. Two thresholds, one for tick population dynamics and the other for Lyme-pathogen transmission dynamics, are identified and shown to fully classify the long-term outcomes of the tick invasion and disease persistence. Seeding with the realistic parameters, the tick reproduction threshold and Lyme disease spread threshold are estimated to illustrate the joint effects of the climate change and host community diversity on the pattern of Lyme disease risk. It is shown that climate warming can amplify the disease risk and slightly change the seasonality of disease risk. Both the “dilution effect” and “amplification effect” are observed by feeding the model with different possible alternative hosts. Therefore, the relationship between the host community biodiversity and disease risk varies, calling for more accurate measurements on the local environment, both biotic and abiotic such as the temperature and the host community composition.

## Introduction

Lyme disease is acknowledged as a common infectious disease for the most of the world, especially in Europe and North America. The disease is caused by a bacterium called *Borrelia burgdorferi*, transmitted by ticks, especially *Ixodes scapularis*[1, 2]. It affects both humans and animals, with more than 30,000 cases reported annually in the United States alone [3]. The pathogen transmission involves three ecological and epidemiological processes: nymphal ticks infected in the previous year appear first; these ticks then transmit the pathogen to their susceptible vertebrate hosts during a feeding period; the next generation larvae acquire infection by sucking recently infected hosts’ blood and these larvae develop into nymphs in the next year to complete the transmission cycle.

Understanding the factors that regulate the abundance and distribution of the Lyme-pathogen is crucial for the effective control and prevention of the disease. Host diversity and temperature variation have direct influence on Lyme transmission patterns [4]. The tick vectors need to complete the transition of four life stages of metamorphosis (eggs, larvae, nymphs and adults) and each postegg stage requires a blood meal from a wide range of host species, and every host species has a specialized reservoir competence, namely ability to carry and transmit the pathogen [5]. Moreover, weather conditions (temperature, rainfall, humidity, for example) are known to affect the reproduction, development, behavior, and population dynamics of the arthropod vectors [6–9], thereby the spread of the Lyme-pathogen in vectors. In particular, the temperature is regarded as an important factor affecting the tick development and tick biting activity, which gives rise to tick seasonal dynamics [1, 10, 11]. In summary, host diversity [12–16], stage structure of ticks [1, 13, 17–22] and climate effects [1, 10, 11, 13, 20, 23] are considered to be crucial for the persistence of Lyme infection. Therefore modeling Lyme-pathogen transmission with multiple tick life stages, tick seasonality and host community composition is pivotal in understanding the pathogen transmission.

**Figure 1:**
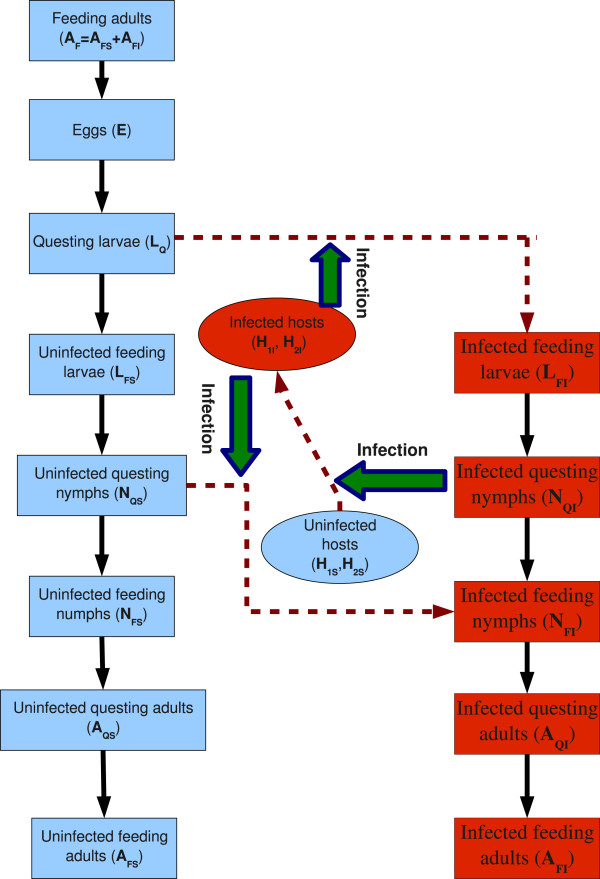
**Schematic diagram for the Lyme disease transmission.** To describe the tick development and biting activities, the tick population is divided into 7 stages, stratified further as the uninfected or infected epidemiological classes for postegg stages. Immature ticks can feed on two host species, the mice (*H*_1_) and an alternative host (*H*_2_), while adult ticks are assumed to feed only on deer in this study.

There have been a range of tick-borne disease modeling efforts dedicating to different aspects of Lyme disease transmission: the basic Lyme transmission ecology [24, 25], effect of different hosts and their densities on the persistence of tick-borne diseases [15, 16, 26], threshold dynamics for disease infection [27, 28], seasonal tick population dynamics and disease transmission [2, 10, 29], climatic effects [1, 2, 22], spatial heterogeneity [18, 30, 31], among others. These previous studies promote our understanding on the transmission mechanisms and designing effective prevention and control measures. In this paper, we develop a modeling framework incorporating the impact of multiple tick life stages, tick seasonality and host diversity on the Lyme disease transmission cycle. We follow the generic model proposed by Randolph and Rogers [21], and divide the vector population into 7 stages with 12 subclasses, as illustrated in Figure 1. This generic model is able to account for the following key features: (i) temperature-dependent/temperature-independent development rates; (ii) temperature-dependent host seeking rates; (iii) density-dependent mortalities, caused by the hosts’ responses during the feeding period; (iv) constant mortalities of off-host development stages. The proposed model below is different from these existing models by incorporating all aforementioned aspects of Lyme disease transmission in a single framework, and as such this framework permits us to analytically define the thresholds of tick population dynamics and pathogen transmission dynamics under seasonal temperature variation, and establish the relationship of these thresholds to the tick establishment and pathogen persistence.

The remaining parts of the paper are organized as follows. In the spirit of striking a delicate balance between the feasibility for the recognized mathematical analysis and the necessity for capturing the key ecological/epidemiological reality, a stage-structured deterministic model is formulated in the next section. Moreover, two thresholds, one for the tick population dynamics and the other for the Lyme-pathogen transmission dynamics are derived and shown to be pivotal in determining the tick population establishment and disease invasion. The model is parameterized in section ‘Model parametrization’. Then the question concerning whether the climate change and an additional host species can amplify/dilute disease prevalence and change the seasonality of disease risk will be addressed through model simulation in section ‘Results’. A discussion section concludes the paper.

## Mathematical model

### Model formulation

In line with the complex physiological process of *Ixodes scapularis*, we divide them into four stages: eggs (*E*), larvae (*L*), nymphs (*N*) and adults (*A*). Each postegg stage is further divided into two groups: questing (*Q*) and feeding (*F*) according to their behavior on or off hosts. Moreover, in terms of their infection status, each group is stratified into two subgroups: susceptible (*S*) and infected (*I*). All variable notations are self-explained as summarized in Table 1. For instance, *L*_*FS*_ represents the subgroup of susceptible feeding larvae.

**Table 1:** **Variable explanations used in the model (1)**

Variable	Meaning
*E*	the number of eggs
*L*_*Q*_	the number of questing larvae
*L*_*FS*_	the number of susceptible feeding larvae
*L*_*FI*_	the number of infected feeding larvae
*N*_*QS*_	the number of susceptible questing nymphs
*N*_*QI*_	the number of infected questing nymphs
*N*_*FS*_	the number of susceptible feeding nymphs
*N*_*FI*_	the number of infected feeding nymphs
*A*_*QS*_	the number of susceptible questing adults
*A*_*QI*_	the number of infected questing adults
*A*_*FS*_	the number of susceptible feeding adults
*A*_*FI*_	the number of infected feeding adults
*H*_1*I*_	the number of infected white-footed mice
*H*_2*I*_	the number of infected alternative hosts

We assume that the host community of the tick population contains three species groups: (i) the white-footed mice *H*_1_ (mainly *Peromyscus leucopus*) with the mortality rate , which is widely known as a primary food provider of immature *I. scapularis* ticks and a key reservoir competent host of *B. burgdorferi* reflecting the strong ability to be infected with the pathogen and to transmit the pathogen to its vector; (ii) the white-tailed deer *D* (mainly *Odocoileus virginianus*), which is believed to be the paramount food provider for adults and in-transmissible for the spread of Lyme-pathogen [32]; and (iii) an alternative host *H*_2_ with mortality rate  such as the eastern chipmunk, the Virginia opossum and the western fence lizard, which is used to study the impact of host community composition on the Lyme disease risk. For the sake of simplicity, we further assume that the total number of each host species (susceptible plus infected) in an isolated habitat is constant. However the number of infected hosts can vary with time, denote by *H*_1*I*_ and *H*_2*I*_, respectively. The Lyme-pathogen transmission cycle between the hosts and multi-stage tick population is presented in the diagram of Figure 1.

In the host-pathogen-tick transmission cycle, larvae and nymphs will bite their host species, however their biting preference to different host species may be different. In order to identify the difference, we use the coefficients, *p*_1_(*p*_2_), to describe larval (nymphal) ticks biting bias on their hosts [33, 34]. Specifically, *p*_1_>1(*p*_2_>1) indicates one host *H*_2_ can attract more larval (nymphal) bites than one host *H*_1_ and vice versa when 0<*p*_1_<1(0<*p*_2_<1). Using the method described in [35],  is the average rate at which a susceptible questing larva finds and attaches successfully onto the infected mice, where *F*_*L*_(*t*) is the feeding rate of larvae, and then  is the average infection rate at which a susceptible larva gets infected from mice, where  is the pathogen transmission probability per bite from infectious mice *H*_1_ to susceptible larvae. Using the same idea, the infection rate of larvae from the infected alternative host *H*_2_ can be accounted. Therefore, the larval infection rate is given by


Similarly, the nymphal infection rate which comes from the contact of questing susceptible nymphs and infectious hosts is given by


The susceptible hosts can get infected when they are bitten by infected questing nymphs. The conservation of bites requires that the numbers of bites made by ticks and received by hosts should be the same. The disease incidence rate for mice is therefore given by


Similarly, the alternative host is infected by the infectious nymphal biting at a rate


Therefore, the disease transmission process between ticks and their hosts can be described by the following system:
1

We assume all the coefficients in the system are nonnegative and the time-dependent coefficients are *τ*-periodic with period *τ*=365 days. The detailed parameter definitions and sample values of these parameters are represented in Table 2. These time-dependent parameters will be estimated in subsection ‘Time-dependent parameters’ below.

**Table 2:** **Definitions and corresponding values of the model parameter with the daily timescale**

Parameter	Meaning	(Value, [reference]) or estimation
*μ*_*E*_	mortality rate of eggs	(0.0025, [1])
*μ*_*QL*_	mortality rate of questing larvae	(0.006, [1])
*μ*_*QN*_	mortality rate of questing nymphs	(0.006, [1])
*μ*_*QA*_	mortality rate of questing adults	(0.006, [1])
*μ*_*FL*_	natural mortality rate of feeding larvae	(0.038, A)
*μ*_*FN*_	natural mortality rate of feeding nymphs	(0.028, A)
*μ*_*FA*_	natural mortality rate of feeding adults	(0.018, A)
*H*_1_	the number of white-footed mice	(200, [1])
	transmission probability from *H*_1_ to larvae	(0.6, [13])
	transmission probability from nymphs to *H*_1_	(1, [13])
	death rate of the white-footed mice	(0.012, [13])
*H*_2_	the number of alternative host *H*_2_	(variable)
	transmission probability from *H*_2_ to larvae	(variable, [36])
	transmission probability from nymphs to *H*_2_	(variable, [36])
	death rate of the alternative host *H*_2_	(variable)
*D*	the number of deer	(20, [1])
*p*	the maximum number of eggs produced	(3000, [1])
*p*_1_	larval biting bias for host *H*_2_	(variable, [37])
*p*_2_	nymphal biting bias for host *H*_2_	(variable, [37])
*b*(*t*)	birth rate of eggs produced	(see subsection ‘Time-dependent parameters’)
*d*_*E*_(*t*)	development rate of eggs	(see subsection ‘Time-dependent parameters’)
*d*_*L*_(*t*)	development rate of larvae	(see subsection ‘Time-dependent parameters’)
*d*_*N*_(*t*)	development rate of nymphs	(see subsection ‘Time-dependent parameters’)
*F*_*L*_(*t*)	feeding rate of larvae	(see subsection ‘Time-dependent parameters’)
*F*_*N*_(*t*)	feeding rate of nymphs	(see subsection ‘Time-dependent parameters’)
*F*_*A*_(*t*)	feeding rate of adults	(see subsection ‘Time-dependent parameters’)
*D*_*L*_	density-dependent mortality rate of feeding larvae	(, E)
*D*_*N*_	density-dependent mortality rate of feeding nymphs	(, E)
*D*_*A*_	density-dependent mortality rate of feeding adults	(, E)

Where *E*: estimation based on [38] and *A*: assumption.

### Dynamics analysis

#### Positivity and boundedness of solutions

Our first task is to show that the mathematical model (1) is biologically meaningful. To do this, we first establish the following theorem to ensure that all solutions through nonnegative initial values remain nonnegative and bounded. You may refer Appendix 1 for the proof.

##### **Theorem2.1**.

For each initial value , system (1) has a unique and bounded solution *x*(*t*,*x*^0^). Moreover, the solution *x*(*t*,*x*^0^)remains in *X* for any *t*≥0. Here,  denotes a generic point with components


Using change of variables *L*_*F*_=*L*_*FS*_+*L*_*FI*_, *N*_*Q*_=*N*_*QS*_+*N*_*QI*_, *N*_*F*_=*N*_*FS*_+*N*_*FI*_, *A*_*Q*_=*A*_*QS*_+*A*_*QI*_ and *A*_*F*_=*A*_*FS*_+*A*_*FI*_, system (1) reduces to
2

Note that we have other three equations for infected feeding nymphs (*N*_*FI*_), questing adults (*A*_*QI*_) and feeding adults (*A*_*FI*_), which is decoupled from the above system. Biologically, we pay attention to the population size of infected questing nymphs whose bites are the main courses of human Lyme disease. We thereby focus on system (2) in the remaining of the paper.

#### The tick population dynamics

We firstly consider the following stage-structured system for the tick population growth decoupled from system (2):
3

Linearization of system (3) at zero leads to the following linear system
4

Let *F*(*t*)=(*f*_*ij*_(*t*))_7×7_, where *f*_1,7_(*t*)=*b*(*t*) and *f*_*i*,*j*_(*t*)=0 if (*i*,*j*)≠(1,7), and *V*(*t*) =


Then we can rewrite (4) as


where a vector *x*(*t*)=(*E*(*t*),*L*_*Q*_(*t*),*L*_*F*_(*t*),*N*_*Q*_(*t*),*N*_*F*_(*t*),*A*_*Q*_(*t*),*A*_*F*_(*t*))^*T*^. Assume *Y*(*t*,*s*), *t*≥*s*, is the evolution operator of the linear periodic system . That is, for each , the 7×7 matrix *Y*(*t*,*s*) satisfies


where *I* is the 7×7 identity matrix. Let *C*_*τ*_ be the Banach space of all *τ*-periodic functions from  to , equipped with the maximum norm. Suppose *ϕ*∈*C*_*τ*_ is the initial distribution of tick individuals in this periodic environment. Then *F*(*s*)*ϕ*(*s*) is the rate of new ticks produced by the initial ticks who were introduced at time *s*, and *Y*(*t*,*s*)*F*(*s*)*ϕ*(*s*) represents the distribution of those ticks who were newly produced at time *s* and remain alive at time *t* for *t*≥*s*. Hence,


is the distribution of accumulative ticks at time *t* produced by all those ticks *ϕ*(*s*) introduced at the previous time.

Following ideas proposed in [39, 40], we define a next generation operator *G*:*C*_*τ*_→*C*_*τ*_ by


Then the spectral radius of *G* is defined as . In what follows, we call  as a threshold for tick population dynamics.

Let *Φ*_*P*_(*t*) and *ρ*(*Φ*_*P*_(*τ*)) be the monodromy matrix of the linear *τ*-periodic system  and the spectral radius of *Φ*_*P*_(*τ*), respectively. Then, from [40], Theorem 2.2, we conclude (i)  if and only if *ρ*(*Φ*_*F*−*V*_(*τ*))=1; (ii)  if and only if *ρ*(*Φ*_*F*−*V*_(*τ*))>1; (iii)  if and only if *ρ*(*Φ*_*F*−*V*_(*τ*))<1. We also know that the zero solution is locally asymptotically stable if , and unstable if .

Note that the Poincar map associated with system (3) is not strongly monotone since some coefficients are not strictly positive (remain zero in a nonempty interval). However, if we regard a *τ*-periodic system (3) as a 6*τ*-periodic system, we can show that the Poincar map with respect to the 6*τ*-periodic system is strongly monotone by using the same idea as in [41], Lemma 3.2. We then use [42], Theorem 2.3.4, to the Poincar map associated with system (3) to obtain the following result, with the proof in Appendix 2.

##### **Theorem2.2**.

The following statements are valid:

(i) If , then zero is globally asymptotically stable for system (3) in ;
(ii) If , then system (3) admits a unique *τ*-positive periodic solution

and it is globally asymptotically stable for system (3) with initial values in .

### The global dynamics of the full model

If threshold for ticks , then there exists a positive periodic solution,


for system (3) such that


In this case, equations for the infected populations in system (2) give rise to the following limiting system:
5

Following ideas of [39, 40], as proceed in the definition of  in the previous section, we can define a threshold for the pathogen. To do this, we introduce


and


Assume (*t*,*s*), *t*≥*s*, is the evolution operator of the linear periodic system . Let  be the Banach space of all *τ*-periodic functions from  to , equipped with the maximum norm. Suppose  is the initial distribution of infectious tick and host individuals in this periodic environment. Then  is the rate of new infectious ticks and host individuals produced by the initial infectious ticks and hosts who were introduced at time *s*, and  represents the distribution of those ticks who were newly produced at time *s* and remain alive at time *t* for *t*≥*s*. Hence,


is the distribution of accumulative infectious ticks and hosts at time *t* produced by all those infectious individuals *ϕ*(*s*) introduced at the previous time. Define the a next generation operator  by


It then follows from [39, 40] that the spectral radius of  is define as , and shows that it is a threshold of the Lyme-pathogen dynamics (5).

Using the same argument as in the proof of Theorem 2.2 (see also the proof of Lemma 2.3 in [43]), we have the following results:

#### **Theorem2.3**.

(i) If , then zero is globally asymptotically stable for system (5) in ; (ii) If , then system (5) admits a unique positive periodic solution  and it is globally asymptotically stable for system (5).

Based on the aforementioned two thresholds,  for ticks dynamics and  for the pathogen dynamics, we can completely determine the global dynamics of the system (2). The detailed proof is shown in Appendix 3.

#### **Theorem2.4**.

Let *x*(*t*,*x*^0^) be the solution of system (2) through *x*^0^. Then the following statements are valid:

(i) If , then zero is globally attractive for system (2);
(ii) If  and , then

and  for *i*∈[8,11];

(iii) If  and , then there exists a positive periodic solution *x*^∗^(*t*), and this periodic solution is globally attractive for system (2) with respect to all positive solutions.

### Summary of mathematical results

By incorporating the tick physiological development and multiple host species, we propose a seasonal deterministic stage-structured Lyme disease transmission model. The model turns out to be a periodic system of ordinary differential equations with high dimensions. As the pathogen has a negligible effect on population dynamics of the ticks and their hosts, the dynamics of the ticks is independent of the pathogen occurrence. This allows us to obtain an independent subsystem for the dynamics of the tick population. Taking the advantage of this observation and with the help of the developed theory for chain transitive sets, we are able to derive two results on global stability of the model system (2). Two biologically significant indices, the tick reproduction threshold  and the Lyme disease invasion threshold  are derived and shown to completely classify the long term outcomes of the tick and pathogen establishment.

## Model parametrization

In this section, we present the estimation of the time-dependent parameters and other parameters related to the host species.

### Alternative hosts species and their reservoir competence

To study the potential effect of of host community biodiversity on the risk of Lyme disease, three types of alternative host species are considered which are different from their reservoir competence, namely, the product of host infection probability bitten by infectious nymphs and larvae infection probability from infectious hosts [36]. The first type is considered as the one with high reservoir competence such as the short-tailed shrew, the marked shrew and the eastern chipmunk. The values of  and  are set as 0.569 and 0.971, respectively, as reported in [36]. The second type that we want to compare is the one with low reservoir competence, in which  and  are set to be 0.0025 and 0.261, respectively, which are similar to those in [36] for the Virginia opossum. The third type of host species is non-competent, ==0, such as the western fence lizard. The authors in [44, 45] stated that the western fence lizard is not able to spread the Lyme-pathogen since the species has a powerful immune system so that it can clean up the Lyme-pathogen when it is bitten by an infected tick. The death rate of each host species is set as  per day due to their similar life spans.

### Time-dependent parameters

In order to investigate the impact of climate warming on the seasonal tick population abundance and Lyme-pathogen invasion, temperature is considered as a variable index in our study and we assume that it changes periodically with time. Therefore, those time-dependent coefficients are indeed temperature-dependent, and periodic in time. In order to parameterize these coefficients, we first estimate these values at a discrete manner at each day of a year, then these coefficients are smoothed into a continuous manner by employing Fourier series. In the remaining of this subsection, we will estimate each time-dependent coefficient at each day of a year.

**Figure 2:**
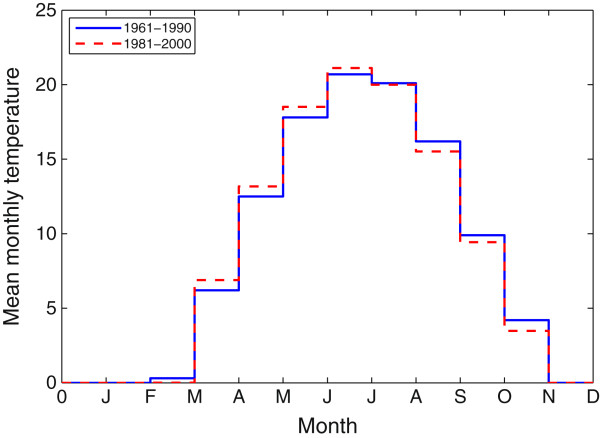
**30 year normal mean monthly temperature under two settings near Long Point.** The blue solid and red dashed curves represent the monthly temperature for the periods 1961−1990 period and 1981−2010, respectively. We set monthly temperature to be 0°C if it is lower than 0°C. Both are collected from Environment Canada website [46].

To begin with this, the model is parameterized for the location Long Point, reported to be the first tick endemic area in Canada. Two temperature datasets for this area are collected from nearby meteorological stations, the Port Dover for the period 1961−1990 and Delhi CDA for the period 1981−2010 due to the unavailability of Port Dover Station recently. For these two stations, the 30-year normal temperature data are collected from the Environment Canada website (Figure 2) [46].

Next we turn to the estimation of time-dependent development rates: *b*(*t*_*i*_), *d*_*E*_(*t*_*i*_), *d*_*L*_(*t*_*i*_) and *d*_*N*_(*t*_*i*_), at day *t*_*i*_ for *t*_*i*_=1,2,⋯,365. To estimate these values, the following relations [1, 8, 47–49]
6789

will be used, where *T*(*t*_*i*_) represents temperature at the specific day *t*_*i*_ in unit Celsius (°C). Using the same method presented in [2], the birth rate *b*(*t*_*i*_) is directly obtained from the product of maximum number of eggs *p* produced and the reciprocal of duration of pre-oviposition period at day *t*_*i*_ as shown Eq. 6, namely *b*(*t*_*i*_)=*p*/*D*_1_(*t*_*i*_). The development rate of eggs *d*_*E*_(*t*_*i*_) is directly calculated as reciprocal of development duration from egg to larva (Eq.7). The calculation of development rate of nymphs *d*_*N*_(*t*_*i*_) is composed by two cases: (i) it is directly estimated as a reciprocal of development duration from nymph to adult (Eq. 9) before diapause; (ii) it is calculated by the method in [2] during diapause. The estimate of larval development rate *d*_*L*_(*t*_*i*_) is a bit complex. We first consider the concept of the daily development proportion of larvae which is calculated as the reciprocal of development duration from larva to nymph at some specific days (Eq. 8) [2]. To obtain *d*_*L*_(*t*_*i*_), we therefore calculate all daily development proportions from day *t*_*i*_ until day *t*_*i*_+*n* for the subsequent *n* days such that the sum of these proportions reaches unity, then *n* is regarded as the development duration of larvae at the specific day *t*_*i*_. Finally, *d*_*L*_(*t*_*i*_) is estimated as  which is dependent of the temperatures of subsequent days.

**Figure 3:**
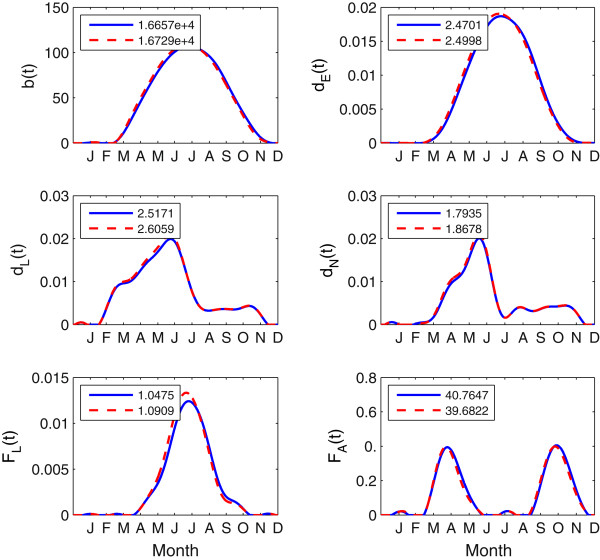
**Development rates and feeding rates of** ***I. scapularis*** **ticks within one year period.** The blue solid and red dashed curves are related to the associated development rates and feeding rates under temperatures in the periods 1961−1990 and 1981−2010, respectively; The numbers at the left top corner in each subfigure indicate the areas under the associated curves, which are used to differentiate the differences of these rates under the two temperature settings.

The feeding rates *F*_*L*_(*t*_*i*_), *F*_*N*_(*t*_*i*_) and *F*_*A*_(*t*_*i*_), affected by both hosts abundance and ambient temperatures, are directly calculated from the following formulas [1]:


where *θ*^*L*^(*T*(*t*_*i*_)), *θ*^*N*^(*T*(*t*_*i*_)) and *θ*^*A*^(*T*(*t*_*i*_)) represent questing activity proportions at respective tick stage at day *t*_*i*_ which are calibrated with data from Public Health Agency of Canada (personal communication). We refer the readers to the literature [2] for more details on the estimation of these periodic parameters. Figure 3 shows the patterns of these time-dependent parameters in one-year for the case *p*_1_= *p*_2_=0.

## Results

We use various indices to measure the Lyme disease risk to humans: (i) , used to determine the tick population persistence; (ii) , as an index for the pathogen population persistence; (iii) density of questing nymphs (DON) in a seasonal pattern; (iv) density of infected questing nymphs (DIN), which reveals the absolute risk of Lyme disease by showing the absolute amount of infected ticks and the pattern of seasonality; and (v) nymphal infection prevalence (NIP) in a seasonal pattern, the proportion of the number of infected questing nymphs in total number of questing nymphs, which characterizes the degree of humans to be infected. All these are widely used indices and we use them to jointly measure the Lyme disease risk to humans [1, 12, 16, 18, 26, 28, 31].

In all simulations, every solution, irrespective of the initial values, of the model system (2) approaches to a seasonal state which is consistent with the theoretical results. Moreover, disease risk goes extinct when , while the seasonal risk pattern appears when . The numerical calculation of  is implemented by the dichotomy method where the system *dX*/*dt*= has a dominant Floquet multiplier equal to 1 [50]. A similar method is used to estimate . In what follows, all results are based on the model outputs at the steady state by running 40 years simulations.

### Impact of climate warming on tick population growth and pathogen transmission

To study the potential effect of climate warming on disease risk, we compare simulations for two different temperature settings, at periods 1961−1990 and 1981−2010, with the absence of alternative host species. The curves of time-dependent parameters under these two temperature settings are shown in Figure 3. Moreover, the numbers on the upper left corner represent the areas under the corresponding curves, reflecting the variation of time-dependent parameters in different temperature consitions. We notice that the development rates and the feeding rates of immature ticks increase with increased temperature. However the feeding rate of adults decreases instead, which is because adult ticks have the limiting host seeking capacity when the temperature is too low or high [1].

**Figure 4:**
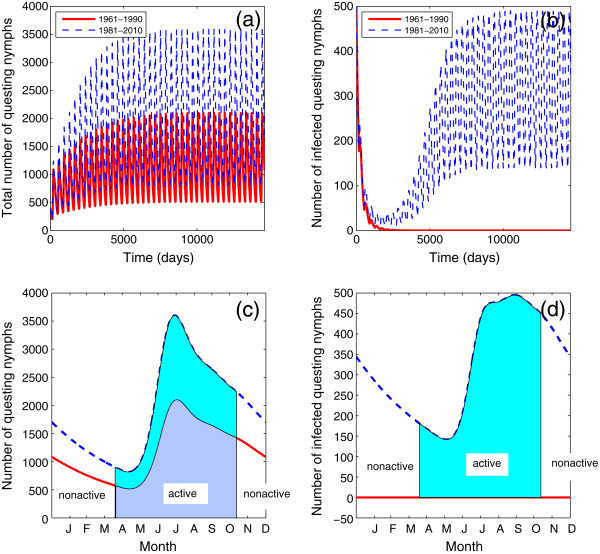
**The variations in the sizes of total questing nymphs and infected questing nymphs with the two temperature settings mentioned above.** The red solid curves represent the outputs by seeding the model with 1961−1990 temperature data ( and  in this case), while the blue dashed curves represent the model outputs by 1981−2010 temperature data ( and  in this case). **(a)** Total questing nymphs; **(b)** infected questing nymphs in the 40 year simulation; **(c)** seasonality of questing nymphs at the steady state; **(d)** seasonality of infected questing nymphs at the steady state, where shaded portions in both **(c)** and **(d)** represent the active seasons of the questing nymphs.

With climate warms up from the period 1961−1990 to 1981−2010, the value of  increases from 1.38 to 1.62, and the values of  also increases from 0.90 (below unity) to 1.19 (above unity). As shown in Figure 4, our simulations confirm the persistence of tick population when  and establishment of pathogen population if . These are in agreement with the theoretical conclusions. We also notice that the number of questing nymphs increases with higher temperature (Figures 4(a), (c)). Moreover, the pattern of infected questing nymphs changes from extinction to an absolutely positive stable oscillation showing the emergence of disease risk (Figures 4(b), (d)). It is important to notice that the active window of (infected) nymphs has been slightly enlarged with warmer temperature (Figure 4(c)). In summary, our study shows that climate warming plays an important role to accelerate the reproduction of the tick population and extend their active windows, and therefore increase the risk of Lyme disease. Moreover, the pattern of seasonality for ticks and pathogens may be changed with the temperature.

### Impact of host biodiversity on the disease risk

Now, we seed the model with temperature condition in the 1981−2010 period so that time-dependent birth rate and development rates remain the same. However, we add an alternative host species to the original host community which is assumed to be composed of the white-footed mice and the white-tailed deer alone. This permits us to study the potential impact of host biodiversity on the risk of Lyme disease. Then, the number of the alternative host species will change the density-dependent death rates and the feeding rates of ticks.

**Figure 5:**
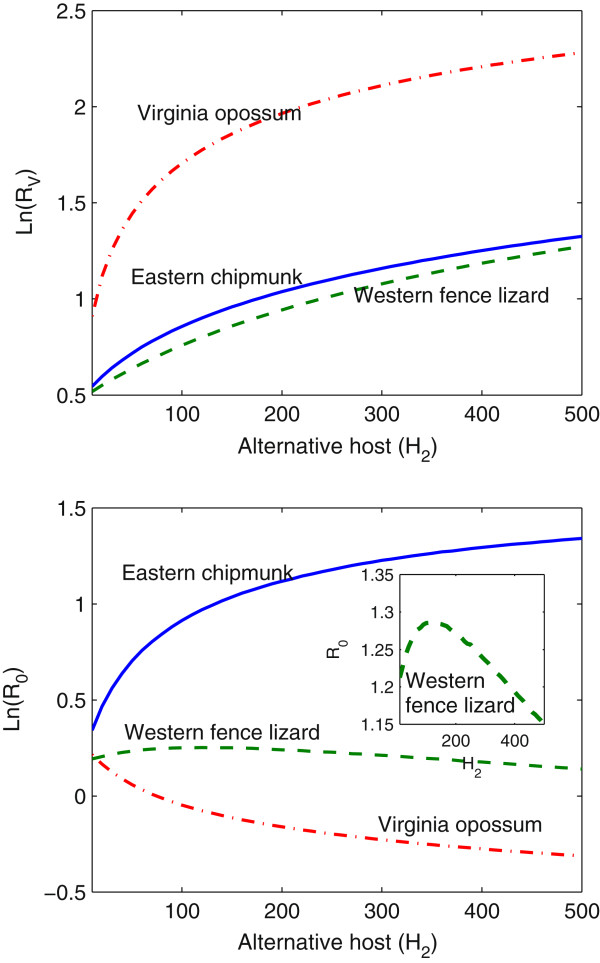
**Log plots of variations of ratios**  **and**  **against the number of alternative hosts.** In the case of the eastern chipmunks, *p*_1_=0.4, *p*_2_=3.5, , , ; for the western fence lizard, *p*_1_=1, *p*_2_=1, , , ; for the Virginia opossum, *p*_1_=7.2, *p*_2_=36.9, , , . For all simulations, the temperature condition is fixed on the period 1981−2010.

As shown in Figure 5, regardless of the newly introduced alternative species, we always observe that the values of  continuously increase with the increased number of hosts; while the change of  is closely connected to the species of the introduced hosts. Introduction of new hosts will always provide more food for the ticks and thus promotes the growth of tick population. However, the variation of the disease risk is not as simple as we imagine. For instance, the values of  persistently increase with the increased number of the eastern chipmunk introduced, however continuously decrease for the Virginia opossum, while first increase then decrease for the western fence lizard (Figure 5). For the eastern chipmunk, recognized as the type with a high reservoir competence ( and ), their ability of Lyme-pathogen transmission and high biting bias coefficient of nymphs (*p*_2_=3.5) facilitate the growth of tick population and spread of the pathogen. For the Virginia opossum with a low reservoir competence ( and ), the reduction of  largely attributes to not only the low transmission ability, but also their large biting biases coefficients (*p*_1_=7.2, *p*_2_=36.9). In this scenario, a great amount of tick bites are attracted to the low competent hosts, and infectious bites are wasted on this incompetent host. For the case of the western fence lizard, we also observe that  increases at the small size of this species even it is a non-competent host, but eventually reduces when the size of western fence lizard attains a certain level.

**Figure 6:**
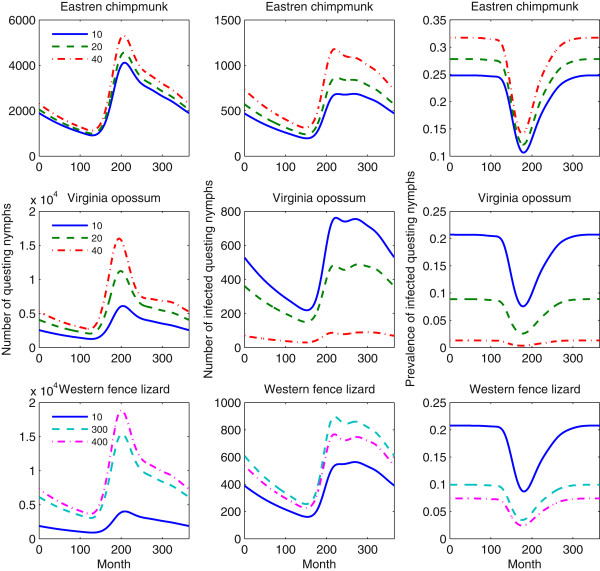
**Variations in the sizes of DON, DIN and NIP under different host sizes.** The numbers on the left panel indicate that the sizes of associated alternative hosts are added into the host community. The scenarios where the eastern chipmunk is added are shown on the upper panel. The middle panel shows the scenarios where the Virginia opossum is considered as the alternative host; the bottom panel shows the situations where the western fence lizard is added. All the associated parameter values are the same as those in Figure 5 except the sizes of alternative hosts.

To clearly understand the “dilute effect” and “amplification effect” in this respect, we would like to examine three indices: DON, DIN and NIP. As shown in Figure 6, introduction of numbers of the eastern chipmunk from 10, 20 to 40 leads to continuous increase of DON, DIN and NIP, and this indicates that the eastern chipmunk offers an efficient host species to amplify the risk of Lyme disease; if the same numbers of the Virginia opossum as these of the eastern chipmunk are added, we notice that DON increases, but both DIN and NIP decrease instead, input of this species indeed reflects the “dilute effect” through reducing not only the absolute amount of infected ticks, but also the proportion of infection; we are surprised to observe that DON continuously increases, DIN first increases and then decreases, while NIP continuously decreases when the western fence lizard is added into the existing host community. That is, this non-competent additional species amplifies the risk of Lyme disease in the sense of absolute amount; on the contrary it also dilutes the risk in the sense of relative proportion of infection. This finding is in good agreement with the debate raised in [14, 51], where authors revealed that the western fence lizard, as a non-competent host, does not always dilute the risk of Lyme disease.

**Figure 7:**
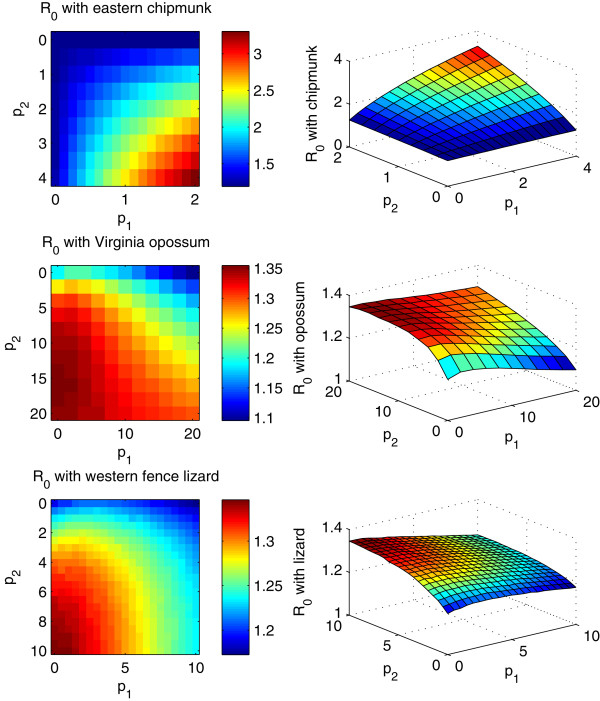
**Relationship between the reproduction ratio**  **,** ***p***_**1**_ **and** ***p***_**2**_ **for the three types of alternative hosts: the eastern chipmunk, the Virginia opossum and the western fence lizard.** The number of alternative hosts is set as 30 and all other parameter values are same as those in Figure 5.

We also perform sensitivity analysis of the threshold  against the biting biases *p*_1_ and *p*_2_. The result shows that  is very sensitive to the variations of both biting biases (Figure 7). Moreover, the relationships between  and *p*_1_ and *p*_2_ varies with host species: (i)  increases with increased *p*_1_ and *p*_2_ in the case of the eastern chipmunk, and therefore this species always facilitates disease transmission within our parameter region; (ii) the relation between  and the larvae bias *p*_1_ is neither positive nor negative for the case of the western fence lizard or the Virginia opossum, which implies both “dilution effect” and “amplification effect” would occur.

## Discussion and conclusion

In this paper, we developed a periodic deterministic system of ordinary differential equations to investigate the impact of both climate condition and host biodiversity on Lyme disease pathogen transmission through the mathematical analysis and computer simulations. The model was parameterized using field and local ecological and epidemiological data. The critical ratios,  and , in combination with other widely used indices, can then provide pivotal information on the impact of temperature variation and host biodiversity on Lyme disease spread.

We found that climate warming facilitates the reproduction of *I. scapularis* population and accelerates the spread of Lyme-pathogen, and then increases the risk of Lyme disease infection. Furthermore, we also have noticed that climate change can slightly change the seasonality of the infected questing nymphs and slightly broaden the active period of the infected questing nymphs, and therefore slightly change the seasonality of the risk of Lyme disease. However, when a new host species was added, we didn’t observe the change of seasonality of the tick population, but we observed the increase of the quantity of total ticks including infected ticks.

The impact of host biodiversity on the Lyme disease risk is a complex issue and remains challenging in conservation ecology and zoonotic epidemiology. However, this issue has both theoretical and practical importance since this may reveal whether the biodiversity conservation can be used as an effective measure for the prevention and control of the zoonotic disease. For Lyme disease, both the dilution effect [5, 52–57] and amplification effect [14] have been observed through field and theoretical studies, where many factors such as spatial scale, host competition, host resistance, tick contact rate were considered [26, 37, 58, 59]. Through this modeling study, both “amplification effect” and “dilution effect” have been observed, where multiple indices (, , DON, DIN and NIP) instead of a single index were utilized. However, the effect does not depend upon the host competence alone, but is a joint outcome of current climate condition, host transmission ability, the numbers of hosts and so on.

In conclusion, climate warming plays a crucial role to speed up the spread of Lyme disease and hence increase the disease risk since climate warming can promote the tick population growth. Introduction of new host species into host community can certainly increase the amount of total ticks, but is not necessary increase the number of infected ticks. In order to obtain a definitive answer to the question “How does the biodiversity of the host community affect the disease risk?”, reliable field study in combination with local abiotic and biotic factors is necessary.

By assuming a spatially homogeneous habitat, the model formulated here has not evaluated the effect of spatial heterogeneity on disease pattern. As ticks can disperse mainly due to its host movement, such as short distance movement due to rodents, long distance travel due to deer [18] and even longer distance because of the bird migration [60]. In 2002, Caraco et al. [18] proposed a reaction-diffusion model for Lyme disease in the northeast United States to investigate the spreading speed of the Lyme disease. The global dynamics of this model was further anlyzed in [61]. A periodic reaction-diffusion system was proposed to study the impact of spatial structure and seasonality on the spreading of the pathogen [31]. The effect of bird migration on Lyme dispersal was studied in [62]. It would be interesting to incorporate our current model formulation into the aforementioned studies involving spatial aspect of Lyme disease spread to address the complicated spatiotemporal spread patterns of Lyme disease with biodiversity and seasonal variation.

## Appendix 1: Proof of Theorem 2.

### *Proof*.

It follows from [63], Theorem 5.2.1, that for any initial value *x*^0^∈*X*, system (1) admits a unique nonnegative solution *x*(*t*,*x*^0^) through this initial value with the maximal interval of existence [0,*σ*) for some *σ*>0.

Let *L*_*F*_=*L*_*FS*_+*L*_*FI*_, *N*_*Q*_=*N*_*QS*_+*N*_*QI*_, *N*_*F*_=*N*_*FS*_+*N*_*FI*_, *A*_*Q*_=*A*_*QS*_+*A*_*QI*_ and *A*_*F*_=*A*_*FS*_+*A*_*FI*_. Then we can see that the tick growth is governed by the following system:
10

For any periodic nonnegative function *f*(*t*) with period *τ*, denote  and . It is easy to see that system (10) can be controlled by the following cooperative system:
11

Clearly, there is only one nonnegative equilibrium zero for system (11) when


If , system (11) admits another positive equilibrium. It then follows from [64], Corollary 3.2, that either zero is globally asymptotically stable or the positive equilibrium is globally asymptotically stable for all nonzero solutions. Hence the comparison principle implies that (*E*(*t*), *L*_*Q*_(*t*), *L*_*F*_(*t*), *N*_*Q*_(*t*), *N*_*F*_(*t*), *A*_*Q*_(*t*), *A*_*F*_(*t*)) is bounded for any *t*∈[0,*σ*). Thus, we see that *σ*=*∞* and the solution for model (1) is bounded and exists globally for any nonnegative initial value. □

## Appendix 2: Proof of Theorem 2.2

### *Proof*.

Theorem 2.3.4 in [42] directly implies that if , then zero is globally asymptotically stable for system (3) in ; if , then system (3) admits a unique 6*τ*-positive periodic solution


and it is globally asymptotically stable for system (3) with initial values in . It remains to prove that the 6*τ*-positive periodic solution (*E*^∗^(*t*), , , , , , ) is also *τ*-periodic. Since for any , = (*E*^∗^(0), , , , , ,  where *P* is the Poincar map associated with the *τ*-periodic system (3). Hence,


On the other hand,


Thus,


which implies that (*E*^∗^(*t*), , , , , , ) is *τ*-periodic. □

## Appendix 3: Proof of Theorem 2.

### *Proof*.

We first consider the *τ*-periodic system as a 11*τ*-periodic system. Let *P* be the Poincar map of system (2), that is, *P*(*x*^0^)=*x*(11*τ*,*x*^0^), where *x*(*t*,*x*^0^) is the solution of system (2) through *x*^0^. Then *P* is compact. Let *ω* = *ω*(*x*^0^) be the omega limit set of *P*(*x*^0^). It then follows from [65], Lemma 2.1, (see also [42], Lemma 1.2.1) that *ω* is an internally chain transitive set for *P*.

(i) In the case where , we obtain  for *i*∈[1,9]. Hence, *ω*= {(0, 0, 0, 0, 0, 0, 0, 0, 0)}×*ω*_1_ for some . It is easy to see that 

where *P*_1_ is the Poincar map associated with the following equation:
12

Since *ω* is an internally chain transitive set for *P*, it easily follows that *ω*_1_ is an internally chain transitive set for *P*_1_. Since {0} is globally asymptotically stable for system (12), [65], Theorem 3.2, implies that *ω*_1_={(0,0)}. Thus, we have *ω*={0}, which proves that every solution converges to zero.

(ii) In the case where , then there exists a positive periodic solution, (*E*^∗^(*t*), , , , , , ), for system (3) such that for any *x*^0^ with , we have 

Thus,  for some , and


where *P*_2_ is the Poincaré map associated with system (5). Since *ω* is an internally chain transitive set for *P*, *ω*_2_ is an internally chain transitive set for *P*_2_. Since , {(0,0,0,0)} is globally asymptotically stable for system (5) according to Theorem 2.3. It then follows from [65], Theorem 3.2, that *ω*_2_={0}. This proves


Therefore, statement (ii) holds.

(iii) In the case where  and , then there exists a positive periodic solution, , for system (3) such that for any *x*^0^ with , we have 

It then follows that  for some , and


where *P*_2_ is the solution semiflow of system (5). Since *ω* is an internally chain transitive set for *P*, it follows that *ω*_3_ is an internally chain transitive set for *P*_2_. We claim that *ω*_3_≠{0} for any .

Assume that, by contradiction, *ω*_3_={0}. That is


for some . Then, we have
13

Since , there exists some *δ*>0 such that the spectral radius of the Poincar map associated with the following linearized system is greater than unity:


It then follows from the same argument as in the proof of Theorem 2.3 that the following system


admits a positive periodic *u*^∗^(*t*) such that


Therefore, there exists some *τ*_0_>0 such that for all *t*>*τ*_0_,


Hence, we conclude that


for all *t*>*τ*_0_. By a standard comparison argument, we have


a contradiction to (13).

Since *ω*_3_≠{0} and the positive periodic solution  is globally asymptotically stable for system (5) in , it follows that


where  is the stable set for (, , , ) with respect to the Poincar map *P*_2_. By [65], Theorem 3.1, we then get


Thus,


and hence, statement (iii) is valid.

At last, using a similar argument as in the proof of Theorem 2.2, we can show that the globally attractive 11*τ*-periodic solution in each case is also *τ*-periodic solution. □
